# The complete chloroplast genome of *Alsophila latebrosa*, a common and widespread tree fern (Cyatheaceae)

**DOI:** 10.1080/23802359.2021.1942262

**Published:** 2021-06-22

**Authors:** Zhen Wang, Ruonan Wang, Limin Lin, Ruisi Liu, Sirui Ma, Yongfeng Hong, Ziqing He, Yingjuan Su, Ting Wang

**Affiliations:** a School of Life Sciences, Sun Yat-sen University, Guangzhou, China; b Research Institute of Sun Yat-sen University in Shenzhen, Shenzhen, China; c College of Life Sciences, South China Agricultural University, Guangzhou, China

**Keywords:** *Alsophila latebrosa*, chloroplast genome, phylogenetic analysis

## Abstract

*Alsophila latebrosa* is a common and widespread tree fern of Cyatheaceae. Its complete chloroplast genome is first assembled and reported with 155,724 bp in length, including a large single copy (LSC) region of 85,800 bp, a small single copy (SSC) region of 21,620 bp, and a pair of inverted repeats (IRs) of 24,152 bp. The genome has 133 genes, including 89 protein-coding genes, 33 tRNA genes, eight rRNA genes and three pseudogenes. Maximum likelihood approach was employed to construct the phylogenetic relationship among ten ferns including *A. latebrosa*. The result showed that *A. latebrosa* was most related to *A. costularis* as a sister group with 100% bootstrap support. The complete chloroplast genome sequences of *A. latebrosa* will provide valuable genomic information to further illuminate phylogenetic classification of Cyatheacea.

*Alsophila latebrosa* Wall. ex Hook. is a tree fern belonging to Cyatheaceae. Its trunk is erect for 3–5 m and sori occur near the fertile pinnule midvein (Zhang et al. 2013). This common and widespread fern grows in forests and secondary forests beside valley streams with altitude 300–1000 m and distributes in China (Hainan and south Yunnan), Cambodia, Indonesia, Malaysia and Thailand (Zhang et al. 2013). Classification of ferns, especially tree ferns, is complex and highly labile. As a well-known family, Cyatheaceae is classified according to morphological characteristics (spores and scales, etc.) (Tryon and Tryon 1982; Christenhusz and Chase 2014) and molecular phylogenetic analysis based on chloroplast DNA data (Conant et al. 1994; Korall et al. 2007). However, there are still disputes in classification and phylogenetic relationship. Hence, acquiring of complete chloroplast genome of *A. latebrosa* will provide more molecular resources and contribute to promote phylogenetic investigation.

*Alsophila latebrosa* was collected from Bawangling in Hainan Island (19°04′05.0″N, 109°08′29.0″E). The specimen is deposited at the Herbarium of Sun Yat-sen University (Hong Yongfeng and email: 903324378@qq.com) with the voucher number as Hong201901. Its fresh leaves were used to extract genomic DNA using Tiangen Plant Genomic DNA Kit (Tiangen Biotech Co., Beijing, China). After DNA fragmentation, a 300 bp-insert library was constructed and sequenced on an Illumina Hiseq 2500 platform (Illumina Inc., San Diego, CA). In total, we approximately obtained 6,709,307 raw reads, which were further filtered and trimmed to finally achieve 6,014,022 high-quality clean data using Trimmomatic v0.32 (Bolger et al. 2014). These clean data were *de novo* assembled to complete chloroplast genome with *A. spinulosa* (GenBank: NC_012818.1) as a reference using Velvet v1.2.07 (Zerbino and Birney 2008). Annotation of the genome was conducted by PGA (Qu et al. 2019) and tRNAscan-SE programs (Lowe and Eddy 1997), then adjusted and confirmed using Geneious v8.1 (Kearse et al. 2012). The sequence is uploaded to NCBI with GenBank number MW620065.

The complete chloroplast genome of *A. latebrosa* is a typical circular molecule with 155,724 bp in length, including a large single copy (LSC) region of 85,800 bp, a small single copy (SSC) region of 21,620 bp, and a pair of inverted repeats (IRs) of 24,152 bp. Its genome has 133 genes, involving in 89 protein-coding genes, 33 tRNA genes, eight rRNA genes and three pseudogenes. GC content is 40.4%, 39.5%, 37.8%, and 43.1% in the genome, LSC, SSC, and IR regions, respectively. Fourteen genes occur as duplicated copies, including four protein-coding genes (*rps12*, *rps7*, *psbA*, and *ycf2*), five tRNA genes (*trnA*-UGC, *trnH*-GUG, *trnI*-GAU, *trnN*-GUU, and *trnR*-ACG), four rRNA genes (*4.5S rRNA*, *5S rRNA*, *16S rRNA*, and *23S rRNA*), and one pseudogene (*trnT*-UGU). Two introns present in three genes (*clpP*, *rps12*, and *ycf3*), while a single intron exists in 14 genes (*trnA*-UGC, *trnG*-UCC, *trnI*-GAU, *trnL*-CAA, *trnV*-UAC, *atpF*, *ndhA*, *ndhB*, *petB*, *petD*, *rpl2*, *rpl16*, *rpoC1*, and *rps16*).

For phylogenetic analysis based on complete chloroplast genome sequences, we constructed a maximum-likelihood tree including *A. latebrosa* and other nine ferns with *Lygodium japonicum* as outgroup using RAxML v.8.2.12 with GTRGAMMAI model and 1000 replicates (Stamatakis 2014) after alignment was conducted through MAFFT (Katoh and Standley 2013). The ML tress showed that *A. latebrosa* was most related to *A. costularis* as a sister group with 100% bootstrap support (Figure 1), while genus *Alsophila* was sister to *Cibotium*. The complete chloroplast genome sequences of *A. latebrosa* will provide valuable genomic information to further illuminate phylogenetic relationship of Cyatheacea.

**Figure 1.**
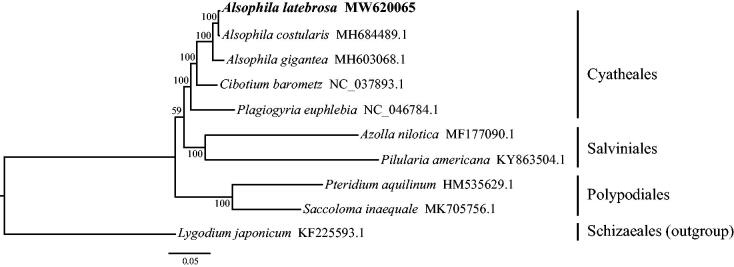
Maximum-likelihood phylogenetic relationship of Alsophila latebrosa and other nine ferns including Lygodium japonicum as outgroup based on whole chloroplast genome sequences. The bootstrap values are shown on each node.

## Data Availability

The data that support the findings of this study are openly available in GenBank of NCBI at https://www.ncbi.nlm.nih.gov/nuccore/MW620065, GenBank accession number MW620065. Raw sequencing reads in this study are deposited in https://www.ncbi.nlm.nih.gov/sra/PRJNA702597, with SRA number SRR13735236.
